# Antimicrobial Efficacy and Stability of an N-Chlorotaurine Gel for Chronic Wound Treatment

**DOI:** 10.3390/ijms26178677

**Published:** 2025-09-05

**Authors:** Zachary M. Thomas, Gabriel J. Staudinger, Sarah E. Hooper, Jeffrey F. Williams, Lori I. Robins

**Affiliations:** 1 Physical Sciences Division, University of Washington Bothell, Bothell, WA 98011, USA; 2 Microbiology & Infection Research Group, Cardiff School of Sport and Health Sciences, Cardiff Metropolitan University, Cardiff CF5 2YB, UK; 3 Briotech Inc., 1102 Shuksan Way, Everett, WA 98203, USA

**Keywords:** biofilms, antimicrobial, gel

## Abstract

The stability of a formulation of 1% N-chlorotaurine (NCT) in a smectite clay as a gel was characterized using a range of physicochemical parameters, and its antimicrobial efficacy was determined against *Staphylococcus aureus* and *Pseudomonas aeruginosa*. The stability of the NCT gel was determined by UV–visible spectroscopy. The efficacy against *S. aureus* and *P. aeruginosa* was tested in single- and dual-species biofilms using a dynamic in vitro chronic wound infection model and only showed efficacy against *S. aureus*. The gel proved stable over time at room temperature and at 4 °C with half-life values of ~161 days and 4 years, respectively. The compatibility of NCT with the preferred pH of the clay gel makes this formulation a candidate for durable topical application to chronic wounds.

## 1. Introduction

N-chlorotaurine (NCT) has emerged as a promising antimicrobial agent since its discovery in 1971 as part of the myeloperoxidase system in innate immunity. It is formed when hypochlorous acid (HOCl), a highly reactive oxidant produced by activated neutrophils, reacts with the amino acid taurine. NCT is considered a long-lived oxidant in vivo that is less reactive than HOCl and participates most often in oxidation reactions with thiols and thioethers. It also chlorinates amines to produce chloramines [1].

Aqueous NCT solutions show broad-spectrum antimicrobial activity in vitro and in various dermatological applications [2,3,4]. We recently demonstrated the stability of aqueous NCT solutions and confirmed their efficacy against both planktonic and biofilm-forming bacteria associated with chronic wound infection [5]. In the context of wound care, these benefits of an NCT solution are offset by limitations such as low viscosity, rapid evaporation, and absorption into dressings, which reduce local bioavailability and contact time. This is particularly problematic in chronic wounds, where biofilms demand prolonged antimicrobial exposure [6].

Here, we report on the analytical characterization and stability of a topical aqueous gel containing 1% NCT in a smectite clay base, a biocompatible carrier commonly used in skin applications and associated with no known contraindications. Our findings confirm high stability with efficacy against *S. aureus* biofilms, highlighting the promise of this formulation for future clinical application.

## 2. Results

**Figure 1:**
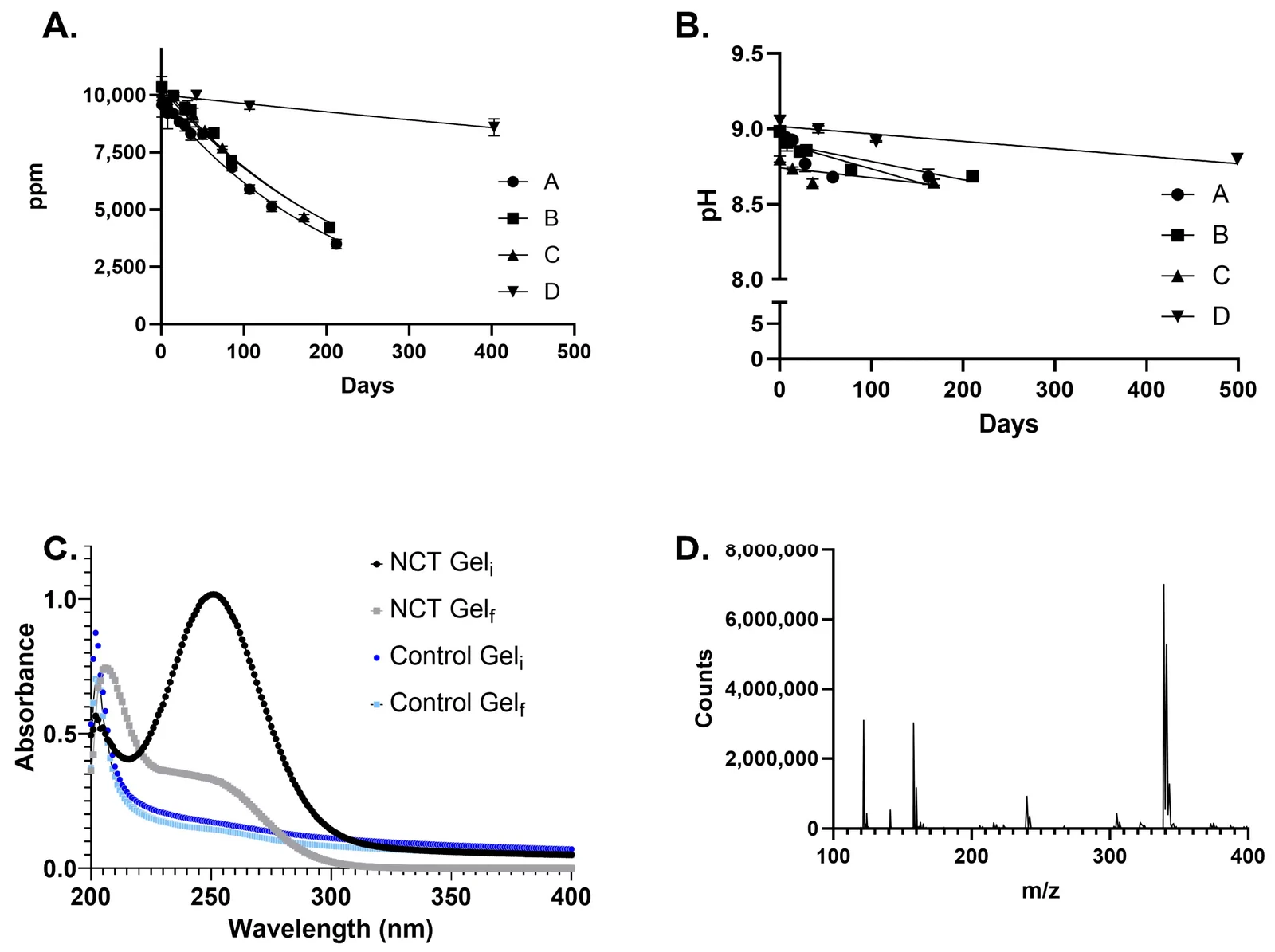
Stability of aqueous 1% NCT gel at ambient temperature and 4 °C. (**A**). Concentration of NCT determined by UV–visible spectroscopy. A–C are triplicates at ambient temperature. D is a triplicate at 4 °C. The mean and standard deviation values are shown with non-linear regression curves fit to the data. (**B**). pH of NCT gel determined for samples at ambient temperature (**A**–**C**) and 4 °C (**D**). (**C**). Representative UV–Vis data for the control and 1% NCT gels at the initial and final time points. (**D**). MS data for the Total Ion Chromatogram (TIC) peak at 252 nm.

The stability of 1% aqueous NCT gels at ambient temperature and at 4 °C was monitored by UV–visible spectroscopy (Figure 1). The NCT concentrations decreased by >50% in the ambient temperature gels, resulting in a half-life of 161 ± 7 days. An average decrease of only 15% occurred after 403 days for the 4 °C gels, and, due to the slow rate of decay, the variability was too large to allow for precise half-life determination. LCMS confirmed that the gel contained only NCT following our previous procedure and identified NCT (157.9684 *m*/*z*), a sodium ion dimer of NCT (338.9261 *m*/*z*), and the elimination imine product (121.9915 *m*/*z*) [5]. The pH of the NCT gel remained constant at ~8.7 for the ambient temperature gels. At 4 °C, the pH was ~9.0. The control gel was constant at pH 11.

**Figure 2:**
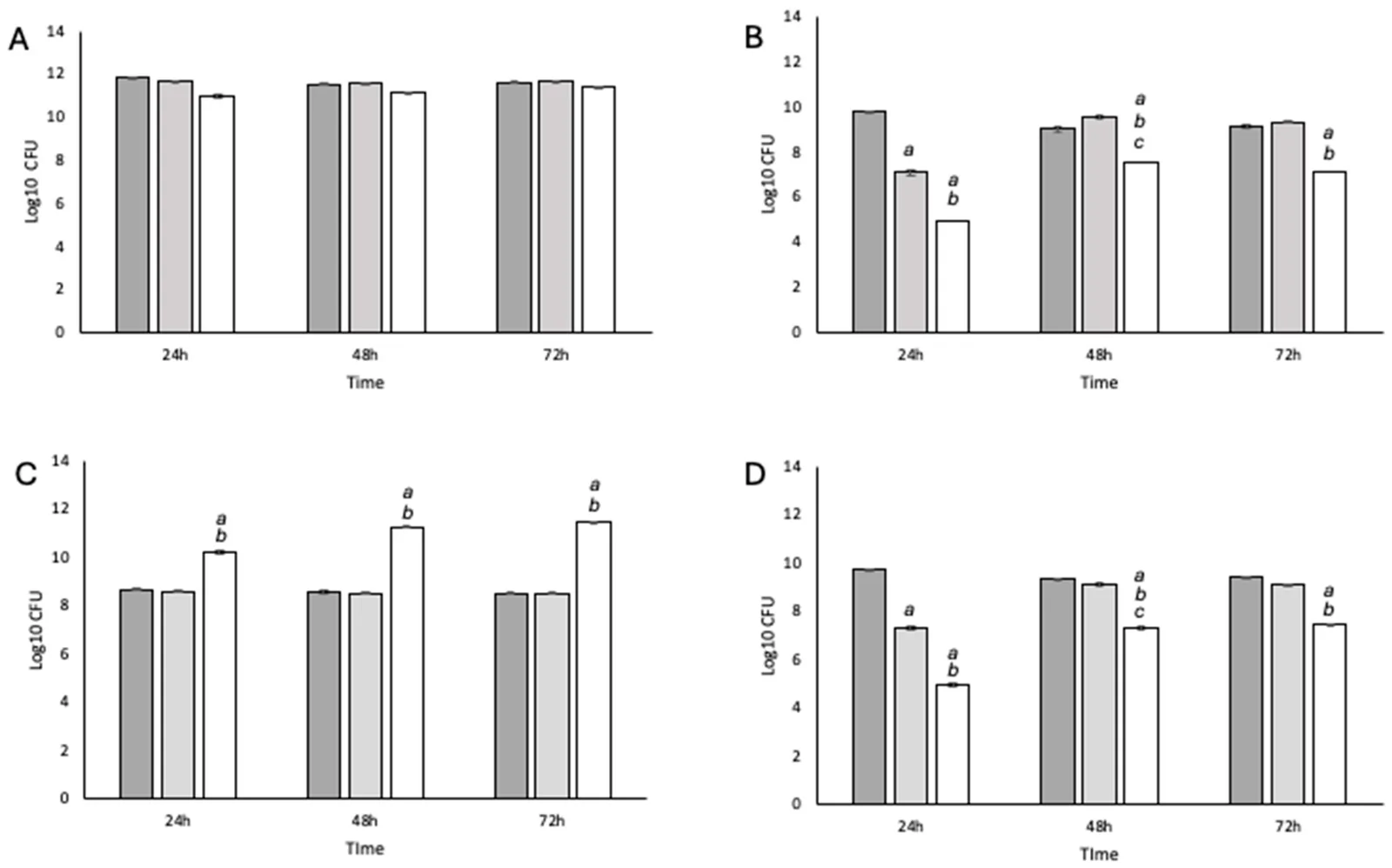
Enumeration of *P. aeruginosa* and *S. aureus* from single- and two-species biofilms. (**A**) *P. aeruginosa* single species; (**B**) *S. aureus* single species; (**C**) *P. aeruginosa* isolated from a two-species biofilm; (**D**) *S. aureus* isolated from a two-species biofilm. Dark gray = untreated control; mid-gray = laponite gel control; white = NCT gel. Statistical significance (*p* < 0.05) and SEM were based on N = 4 and n = 3 and are given as follows: (a) an untreated control compared to laponite gel or NCT gel; (b) a laponite gel compared to NCT gel; (c) T = 24 compared to T = 48.

The efficacy of the NCT gel was tested in single- and two-species biofilms (Figure 2). In both cases, the NCT gel exhibited antimicrobial effects against *S. aureus* but not *P. aeruginosa*. At 24 h, the control gel (pH 11) resulted in a significant (*p* < 0.05) reduction in *S. aureus* in both single and two-species biofilms, but the CFU of the control gel matched the untreated control after 48 h and 72 h (Figure 2B,D). This was not detected with the *P. aeruginosa* biofilms. No significant change was detected in the *P. aeruginosa* single-species biofilm. In the two-species biofilm, numbers of *P. aeruginosa* were significantly higher at all time points tested (*p* < 0.05) compared with the untreated control and the control gel (Figure 2A,C).

## 3. Discussion

The clinical effectiveness of topical antimicrobial agents in chronic wounds is closely linked to the delivery vehicle, which impacts the concentration and duration of antimicrobial exposure [6]. The half-life of our NCT gel at ambient temperature proved to be slightly higher than that of the 1% aqueous NCT solution, with increased stability at 4 °C, as confirmed by LCMS. Both formulations maintain a pH with a range of pH 8.7–9.0. NCT solutions (1%, pH ~8.3) were shown to be effective at reducing pain and decreasing the number of chronic leg ulcers testing positive for the presence of bacteria by 33% in a randomized clinical trial [3,5]. An NCT aqueous gel offers a stable active chlorine solution at high concentrations in contrast to an aqueous active chlorine gel containing HOCl [7].

In agreement with our prior studies, 1% aqueous NCT is understood to have bactericidal activity against planktonic cultures and static biofilms of *S. aureus* and *P. aeruginosa* [5,8]. This investigation allowed us to test a 1% NCT gel using a 3D biofilm model, incorporating both single- and dual-species biofilms to mimic the chronic wound environment [9]. The gel formulation allowed the sample to remain on the biofilm over the testing period, unlike liquid samples that, in our experience, evaporate after 24 h in this model. Although outside the scope of this paper, additional work characterizing NCT gels at varying viscosities may provide insight into the minimum viscosity necessary to prevent evaporation and allow for a more direct comparison to a liquid sample.

The results of our studies indicate that NCT in gel form is more effective against *S. aureus* than *P. aeruginosa*, showing a consistent trend with our previous findings where the Minimum Biofilm Eradication Concentration (MBEC) for *P. aeruginosa* was twice that of *S. aureus* [5]. Reports on the efficacy of aqueous solutions of NCT against single species *S. aureus* and *P. aeruginosa* biofilms show that high concentrations of NCT (1%) are effective against *P. aeruginosa*, while low concentrations are not [10,11]. It is well understood that different biofilm models lead to differences in extracellular polymeric substances (EPS) [12]. In our 3D biofilm model, where bacteria form spatially distinct aggregates encased in EPS, it is possible that the observed difference in susceptibility between *S. aureus* and *P. aeruginosa* is linked to the variation in the EPS [13]. N-acetylglucosamine (GlcNAc), for example, is a key component of polysaccharide intercellular adhesin (PIA) and is essential for *S. aureus* biofilm stability; it is absent in *P. aeruginosa* biofilms. Previous evidence indicates that NCT disrupts the extracellular matrix of *S. aureus* biofilms [10]. It is possible that NCT promotes the degradation of the EPS via direct chlorination of N-acetyl groups like HOCl or by impacting bacterial adherence [10,14,15].

In dual-species biofilms additional complexity is introduced with the potential for bacterial interactions to impact susceptibility and survival. Under these conditions, *P. aeruginosa* treated with the NCT gel significantly increased in number (*p* < 0.05) at all time points compared to controls and the control gel. However, there was no significant difference (*p* > 0.05) in bacterial counts between single- or co-culture models treated with NCT, suggesting bacterial interactions do not affect antimicrobial efficacy. Controls in the two-species biofilm showed 2–3 log fewer *P. aeruginosa* compared to monoculture, indicative of competitive interactions between the bacteria. The higher *P. aeruginosa* numbers when treated with NCT may result from a growth advantage following *S. aureus* elimination and release of growth-promoting nutrients [16]. At 24 h, the laponite vehicle gel significantly reduced *S. aureus* numbers, likely due to its high pH [17,18]. The agarose matrices allow for fluid replacement every 7.6 min, removing the initial inhibitory effects of high pH against *S. aureus*, promoting the recovery growth we observe at 48 h and 72 h [9].

Our results highlight the need for multiple, complementary active chlorine treatments. HOCl and NCT both exhibited antimicrobial efficacy in our model; however, the initial selectivity against *P. aeruginosa* was different [19]. This aligns with previous reports on the reactivity of these two compounds [20,21]. Underpinned by our prior data, these findings support a potential combination strategy in wound care where initial multi-dose application of HOCl for wound cleansing could reduce *P. aeruginosa*, *S. aureus*, and other bacteria associated with chronicity [9,13,19]. Subsequent treatment with a more stable NCT gel could provide sustained antimicrobial activity against *S. aureus*, and potentially other wound bacteria, between dressing changes. This is clinically important, as *S. aureus* in wounds can cause invasive infection [22]. This sequential approach reflects the innate immune response, where neutrophils produce HOCl during the oxidative burst followed by longer-lived chloramines such as NCT, and may offer a practical strategy for managing chronic wounds. Beyond its antimicrobial activity, NCT also reduces inflammation through inhibition of the pro-inflammatory cytokine IL-6, offering additional therapeutic value in wound care [23,24].

## 4. Materials and Methods

### 4.1. Reagents

Chloramine-T trihydrate 98%, taurine 99%, ethyl alcohol 99.5+%, ammonium acetate, and acetonitrile (HPLC grade) were purchased from Thermo Fisher Scientific (Waltham, MA, USA). Laponite XL21 was purchased from BYK USA Inc. (Wallingford, CT, USA). Water was obtained from a Milli-Q water purification system.

Synthesis of NCT: NCT was synthesized as a crystalline sodium salt following our previous procedures [5].

Preparation of aqueous 1% NCT gel: The NCT gel was made following procedures for an HOCl gel [25]. Briefly, laponite (3%) was added to milli-Q water, and the solution was mixed with an emulsion blender for 20–25 min until the sample was homogeneous. A 25% NCT solution in milli-Q water was dissolved in water and added to the homogeneous mixture to make the 1% NCT gel. The sample was further mixed with the emulsion blender for 15–20 min until homogeneous. Samples were stored in 50 mL aliquots at ambient temperature and at 4 °C protected from light. Control gels were made following the same procedure without the addition of NCT; 0.5 mL of 1 M NaOH was added to solidify the control gels.

UV–Vis spectroscopy: The gel (1 mL) was added to 24 mL of 1% NaCl and shaken to disrupt the gel matrix. The sample was diluted into milli-Q water for analysis. The spectrophotometer was blanked with Milli-Q prior to use, and wavelength scans (200–400 nm) were collected using a Thermo-Scientific BioMate 3S. All samples were measured at 252 nm and diluted to fall within the linear range. The extinction coefficient used for NCT was 395.5 M^−1^·cm^−1^ [1].

Determination of NCT gel pH: The gel (1 mL) was added to 24 mL of 1% NaCl and shaken to disrupt the gel matrix. The sample was diluted into milli-Q water for analysis. pH probe was calibrated with pH 7 and 10 standards. The pH data were collected using Fisher Scientific Accumet AB 150 pH benchtop meter. All samples were measured using 5 mL of solution in a small beaker.

Liquid chromatography–mass spectroscopy (LCMS): The NCT gel was diluted 1:2 with milli-Q water and vortexed for 30 s and then centrifuged at 10,000 RPM for 10 min (Beckman Coulter Microfuge 20R, Beckman Coulter, Indianapolis, IN, USA). The supernatant was removed and filtered through a 0.45 µm syringe filter. The solution was centrifuged at 10,000 RPM for 5 min, and the supernatant (3.0 μL) was used for LCMS analysis. An Agilent 1290 Infinity UHPLC system (Agilent, Santa Clara, CA, USA) coupled to an Agilent 6230B time-of-flight mass spectrometer (TOF-MS) (Agilent) equipped with a dual Agilent Jet Stream electrospray ionization source (Agilent) was used for compound separation and analysis. The separation was performed with a ZORBAX RRHD Eclipse Plus C18 column (50 mm × 2.1 mm i.d., 95 Å, 1.8 μm) using 50% (*v*/*v*) 50/50 of 50 mM ammonium acetate (pH = 6.8) and acetonitrile at a 300 μL/min flow rate for 4 min and a column temperature of 20 °C.

The TOF-MS instrument was operated in negative ionization mode with the following settings: 325 °C drying gas temperature with an 8 L/min flow rate, 350 °C sheath gas temperature with an 11 L/min flow rate, and 30 psig nebulizer pressure. Voltage parameters were set as follows: 3500 V capillary, 65 V skimmer, 1000 V nozzle, and 175 V fragmentor.

The data acquisition was performed in the standard mass range (≤*m*/*z* 3200) with 2 GHz extended dynamic range mode at an acquisition rate of 1.0 spectra/s. Data were analyzed using Agilent Qualitative Analysis software (v 10.0). Spectral peak data were background subtracted using pre-injection data.

### 4.2. Bacterial Strains and Culture Conditions

*P. aeruginosa* ATCC 9027 and *S. aureus* NCTC 13616 (EMRSA-15) were cultured in simulated wound fluid (SWF; 2.34 mM CaCl_2_·2H_2_O, 3.75 mM KCl, 9.9 mM NaCl; pH 7.4) containing heat-inactivated fetal bovine serum (FBS; 3% *v*/*v*) and equilibrated to 1 × 10^8^ CFU/mL each for single and mixed biofilms. Biofilms were established in agarose-collagen matrices to mimic the chronic wound environment.

### 4.3. Biofilm Flow System

Biofilms were cultured in a custom flow system maintained at 33 °C with a flow rate of 0.322 mL/min using our previously optimized and published system (for complete protocol see [13]; downloadable procedures including equipment and reagents, relevant references, and detailed model schematics are available at Hooper Group: Biofilm Models). At T0, 0.15 g of NCT gel or laponite gel was applied to the biofilm surface. After 24, 48, and 72 h, biofilms were harvested, washed with PBS to remove loosely adherent organisms, and homogenized in 0.1% (*w*/*v*) sodium thiosulphate to quench residual NCT. Three replicate biofilms for each time point were enumerated using the Miles Misra method using cetrimide agar to isolate *P. aeruginosa* and Baird Parker agar to isolate *S. aureus* [26]. Data comprise four biological replicates, and statistical significance was determined using 2-way ANOVA and Tukey’s post hoc test.

## Data Availability

The original contributions presented in this study are included in the article. Further inquiries can be directed to the corresponding author.
